# Replication Fork Reversal and Protection

**DOI:** 10.3389/fcell.2021.670392

**Published:** 2021-05-10

**Authors:** Shan Qiu, Guixing Jiang, Liping Cao, Jun Huang

**Affiliations:** ^1^Department of General Surgery, Sir Run Run Shaw Hospital, Zhejiang University School of Medicine, Hangzhou, China; ^2^The MOE Key Laboratory of Biosystems Homeostasis and Protection, Zhejiang Provincial Key Laboratory for Cancer Molecular Cell Biology and Innovation Center for Cell Signaling Network, Life Sciences Institute, Zhejiang University, Hangzhou, China; ^3^Zhejiang University-University of Edinburgh Institute, Zhejiang University School of Medicine, Zhejiang University, Haining, China

**Keywords:** replication stress, replication fork stalling, genome instability, replication fork reversal, DNA translocase

## Abstract

During genome replication, replication forks often encounter obstacles that impede their progression. Arrested forks are unstable structures that can give rise to collapse and rearrange if they are not properly processed and restarted. Replication fork reversal is a critical protective mechanism in higher eukaryotic cells in response to replication stress, in which forks reverse their direction to form a Holliday junction-like structure. The reversed replication forks are protected from nuclease degradation by DNA damage repair proteins, such as BRCA1, BRCA2, and RAD51. Some of these molecules work cooperatively, while others have unique functions. Once the stress is resolved, the replication forks can restart with the help of enzymes, including human RECQ1 helicase, but restart will not be considered here. Here, we review research on the key factors and mechanisms required for the remodeling and protection of stalled replication forks in mammalian cells.

## Introduction

Faithful DNA replication during each cell cycle is essential for maintaining genome stability (Jeggo et al., 2016). However, the DNA replication process is frequently challenged by endogenous and exogenous sources of genotoxic stress, including DNA lesions, difficult to replicate sequences, and nucleotide depletion (Mehta and Haber, 2014; Kitao et al., 2018). These challenges, if not properly addressed, would ultimately cause genome instability, a hallmark of tumorigenesis (Jackson and Bartek, 2009; Ou and Schumacher, 2018). Fortunately, organisms have evolved multiple DNA damage repair pathways and DNA damage tolerance (DDT) mechanisms to maintain genome stability (Friedberg, 2005; Huen and Chen, 2010; Branzei and Psakhye, 2016).

DNA damage tolerance refers to the bypassing of DNA lesions and replication restart after the replication fork stalls (Friedberg, 2005). One mode of DDT is replication fork reversal. Proposed in 1976, replication fork reversal was long regarded as a pathological result of fork destabilization, but has now been accepted as a DDT based on recent observations of reversed fork structures *in vivo* and the identification of molecules involved in fork regression *in vitro* (Sakaguchi et al., 2009; Bermejo et al., 2011; Neelsen and Lopes, 2015; Berti et al., 2020a). Emerging evidence suggests that replication fork reversal is indispensable for maintaining genome stability in higher eukaryotic cells. For example, it actively slows down replication fork progression via multiple enzymes, such as the recombinase RAD51 and DNA translocase helicase-like transcription factor (HLTF), which provides sufficient time for the DNA repair machinery to become involved and prevent double-strand break (DSB) formation (Poole and Cortez, 2017; Tye et al., 2020). Replication fork reversal also triggers template switching, where the nascent strand is used for error-free DNA synthesis (Zellweger et al., 2015). However, reversal can render replication forks susceptible to nucleolytic attack (Liao et al., 2018; Rickman and Smogorzewska, 2019). Recent studies have explored factors that can protect reversed forks against nuclease processing, like BRCA1, BRCA2, and components of the Fanconi anemia (FA) complex (Rickman and Smogorzewska, 2019; Tye et al., 2020).

This review focuses on the process of replication fork reversal, especially the enzymes, and molecules involved. First, changes in the replication fork structure after damage blockage, and the factors that promote fork regression, are summarized. The review then explores several mechanisms that protect the reversed fork structure. We hope that this review will provide comprehensive insight into replication fork reversal, thereby contributing to future therapies for diseases like cancers.

## A Two-Step Mechanism for Replication Fork Reversal

In response to replication perturbation, the DNA fork structure changes depending on the type of damage. If a lesion occurs on the lagging strand, it will likely be bypassed because the semi-discontinuous characteristics of DNA replication allow the lagging strand to leave a single strand DNA (ssDNA) gap to be repaired afterward (McInerney and O’Donnell, 2004). However, if a lesion occurs on the leading strand, the fork structure will be altered. In this case, synthesis of the leading strand is inhibited at the blockage point due to polymerase dissociation (also called fork uncoupling), while the helicase continues to generate ssDNA for hundreds of bases (Atkinson and McGlynn, 2009; Berti et al., 2020a). Thus, stalling the synthesis of the leading strand results in an accumulation of ssDNA; this provides a platform for loading multiple enzymes, thereby promoting fork remodeling (Kolinjivadi et al., 2017).

### PCNA Polyubiquitination and Fork Slowing

Proliferation cell nuclear antigen (PCNA) is a highly conserved homotrimer that serves as a DNA clamp and is crucial for DNA replication and associated processes (Boehm et al., 2016; Lee and Park, 2020). It is a critical regulator of DDT, in which PCNA monoubiquitination at lysine 164 (PCNA-Ub) facilitates error-prone translesion DNA synthesis and PCNA polyubiquitination (PCNA-Ub^*n*^) promotes error-free damage bypass (Sale, 2013; Branzei and Szakal, 2017). In yeast, PCNA-Ub^*n*^ is mediated by E3 ubiquitin ligase Rad5, while in mammalian cells it is mediated by the Rad5 orthologs HLTF and SNF2 histone linker PHD RING helicase (SHPRH; Unk et al., 2010). Surprisingly, PCNA-Ub^*n*^ occurs in *Hltf/Shprh* double-deficient mouse embryonic fibroblasts (Krijger et al., 2011). Therefore, another E3 ligase must contribute to PCNA-Ub^*n*^ in mammalian cells. A recent *in vitro* study found that the HECT-type E3 ligase HECW2 interacted with PCNA and regulated its ubiquitination; its role in DDT needs further study (Krishnamoorthy et al., 2018). Strikingly, a recent study demonstrated that K63-linked, UBC13-dependent PCNA-Ub^*n*^ is required to slow and reverse replication forks in response to replication stress (Vujanovic et al., 2017).

### Critical Enzymes in Fork Slowing and Reversal

**FIGURE 1:**
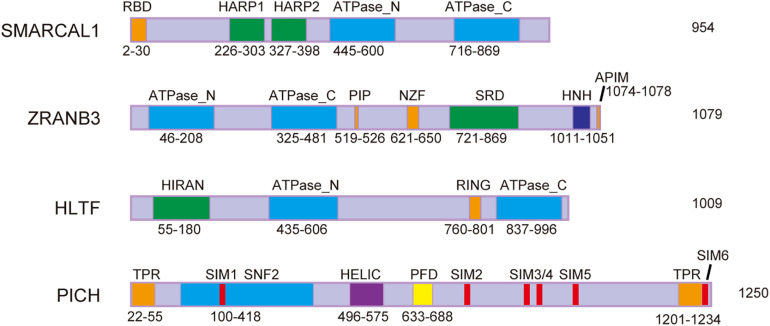
Schematic representation of protein domains of SMARCAL1, ZRANB3, HLTF, and PICH. RBD, RPA-binding domain; HARP, HepA-related protein; PIP, PCNA-interacting protein box; NZF, Npl4 zinc-finger; SRD, substrate recognition domain; HNH, His-Asn-His protein; APIM, AlkB homolog 2 PCNA interacting motif; HIRAN, HIP116 and RAD5 *N*-terminal; RING, really interesting new gene; TPR, tetratricopeptide repeat; SIM, SUMO-interacting motif; SNF2, sucrose non-fermenting 2; HELIC, helicase superfamily *c*-terminal domain; and PFD, PICH family domain.

Emerging evidence suggests that active replication fork slowing upon genotoxic stress is linked to replication fork reversal, which is at least partly regulated by SNF2 family chromatin remodelers, including SMARCAL1 (SWI/SNF-related, matrix-associated, actin-dependent, regulator of chromatin, and subfamily A-like 1), ZRANB3 (zinc finger, RAN-binding domain containing 3), and HLTF (Poole and Cortez, 2017; Figure 1). Mutations in *SMARCAL1* lead to Schimke immuno-osseous dysplasia (SIOD), while *HLTF/ZRANB3*-deficient cells are vulnerable to replication stress and contribute to tumorigenesis (Ciccia et al., 2009; Li et al., 2009; Weston et al., 2012; Helmer et al., 2019). Therefore, these helicase-like proteins play critical roles in DDT, and use energy from ATP hydrolysis to remodel chromatin structure (Hargreaves and Crabtree, 2011). They are recruited to the stalled replication forks by interactions with other proteins, like RPA or PCNA, and then bind DNA sequences via substrate-recognition domains. All three of these DNA translocases can catalyze replication fork regression both *in vitro* and *in vivo*, and have specific, distinct functions in fork remodeling (Blastyak et al., 2010; Achar et al., 2011; Betous et al., 2012).

SWI/SNF-related, matrix-associated, actin-dependent, regulator of chromatin, and subfamily A-like 1 is an annealing helicase that contains a replication protein A (RPA) binding domain. RPA, a eukaryotic ssDNA-binding protein that regulates various DNA metabolic processes, is required for SMARCAL1 localization to stalled forks (Ciccia et al., 2009; Yuan et al., 2009; Byrne and Oakley, 2019). SMARCAL1 interacts with RPA and catalyzes replication fork regression, which is regulated by the ATM and Rad3-related (ATR) protein kinase (Couch et al., 2013; Bhat and Cortez, 2018). While RPA stimulates SMARCAL1 fork reversal activity when it is bound to a ssDNA gap on the leading template strand, it inhibits SMARCAL1 when bound to a replication fork with a ssDNA gap on the lagging strand (Betous et al., 2013).

Zinc finger, RAN-binding domain containing 3 contains a PCNA-interacting protein box and an AIkB homology 2 PCNA interaction motif (APIM) to bind PCNA, which facilitates its localization to stalled forks (Ciccia et al., 2012; Weston et al., 2012; Yuan et al., 2012). Moreover, its NPL4 zinc-finger motif preferentially interacts with K63-linked polyubiquitinated PCNA and is also required for the localization of ZRANB3 at stalled replication forks (Vujanovic et al., 2017). Because of its homologous sequence, ZRANB3 has functions similar to SMARCAL1, including annealing complementary DNA strands and catalyzing fork reversal. Unlike SMARCAL1, however, RPA inhibits the fork reversal ability of ZRANB3 on the leading-strand gaps substrates (Betous et al., 2013). Moreover, unlike other SNF2 family proteins, ZRANB3 exhibits structure-specific ATP-dependent endonuclease activity and can cleave fork DNA structures (Weston et al., 2012). Exactly how these enzymatic activities work together at stalled replication forks remains unknown.

Similar to SMARCAL1 and ZRANB3, HLTF can catalyze fork reversal via ATP hydrolysis. HLTF binds the leading strand via its *N*-terminal HIRAN domain to stimulate fork regression (Achar et al., 2015; Kile et al., 2015). In addition, it has been reported that HLTF partly counteracts the activity of the DNA helicase FANCJ at stalled forks to maintain fork remodeling and prevent unlimited replication (Peng et al., 2018). Unlike the other two DNA translocases, no protein interaction motifs have been discovered in HLTF, and how it is recruited to stalled forks requires further investigation. Although a study has demonstrated that RPA and Pax transactivation domain-interacting protein interacts with HLTF, future research should examine their roles in replication stress (MacKay et al., 2009). Since simultaneously depletion of SMARCAL1, ZRANB3, and HLTF did not show an additive effect on reversed fork frequency, these three DNA translocases may function at different stages of a common pathway (Taglialatela et al., 2017; Tian et al., 2021). It is also possible that each translocase works preferentially on specific substrates or genomic regions, which need further investigation (Taglialatela et al., 2017; Tian et al., 2021).

In addition to the SNF2 family proteins, it has been reported that RAD51 is required for replication fork regression. RAD51 is a highly conserved DNA recombinase that facilitates DNA DSB repair in vertebrates by promoting homologous recombination repair (Gachechiladze et al., 2017; Laurini et al., 2020; Sinha et al., 2020). A nascent chromatin capture screening study detected RAD51 on the replication forks (Alabert et al., 2014). Unlike homologous recombination repair, RAD51 has a non-canonical function in fork reversal, since BRCA2-modulated stable RAD51 filaments are not needed in this process (Bhat and Cortez, 2018). Although the mechanisms are not clear, it has been suggested that RAD51 paralogs (RAD51B, RAD51C, RAD51D, XRCC2, and XRCC3) may assist RAD51 and DNA translocases in promoting replication fork reversal (Berti et al., 2020b). The loaders and specific role of RAD51 in fork reversal warrant further investigation.

Other enzymes have also been reported to participate in the reversal of replication forks. For example, the branch point translocase FANCM (Fanconi anemia complementation group M) could convert a replication fork from a three-way junction to a four-way junction in an ATP-dependent manner (Gari et al., 2008). Moreover, a study showed that FBH1 (F-box DNA helicase 1) was recruited to the stalled forks and could unwind the lagging strands (Masuda-Ozawa et al., 2013). A more recent study demonstrated that the helicase activity of FBH1 was involved in replication fork regression, which was also dependent on ATP hydrolysis (Fugger et al., 2015). Although many related enzymes and molecules have been discovered, it is not clear whether these proteins work together to promote fork remodeling, or if they work independently in response to different replication obstacles. It will be necessary to explore the interactions among these enzymes in the future.

Although the above enzymes play significant roles in replication fork remodeling, they must be tightly regulated as too little or too much of their activities at stalled forks is deleterious for genomic stability. For example, ATR phosphorylates SMARCAL1 at Ser652 to limit its fork regression activity, thereby preventing replication fork collapse (Couch et al., 2013). Apart from ATR, RAD52 also limits SMARCAL1 activity at stalled forks by counteracting its loading (Malacaria et al., 2019). Moreover, the RPA-like single-strand DNA binding protein RADX antagonizes RAD51 filament formation to prevent inappropriate replication fork reversal (Dungrawala et al., 2017; Schubert et al., 2017; Zhang et al., 2020; Adolph et al., 2021).

### The ZATT-TOP2A-PICH Axis and Extensive Replication Fork Reversal

**FIGURE 2:**
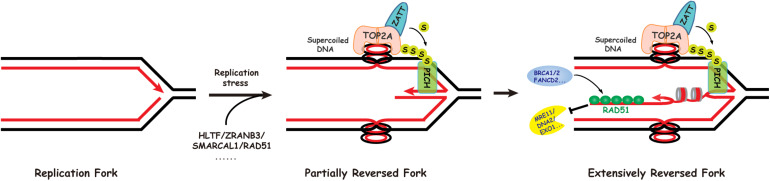
Replication fork reversal occurs via a two-step mechanism. In the first step, SMARCAL1, HLTF, and ZRANB3 cooperate with RAD51 to initiate limited replication fork reversal, generating positive superhelical strain in the newly replicated sister chromatids. The initial fork reversal may be helped by the positive supercoiling ahead of the replication fork created during replication. In the second step, DNA topoisomerase IIalpha (TOP2A) promotes extensive fork reversal, on one hand through resolving the resulting topological barriers, and on the other hand via its role in recruiting the SUMO-targeted DNA translocase PICH to stalled replication forks.

Extrusion of the leading and lagging strands from the template DNA during replication fork reversal, catalyzed by the above enzymes, would cause positive superhelical strain in the newly synthesized sister chromatids (Tian et al., 2021). The resulting superhelical strain prevents further regression of the stalled replication forks and must be dissipated by DNA topoisomerases for reversal to proceed efficiently (Tian et al., 2021). Our recent study found that DNA topoisomerase 2 (mainly TOP2A) can release the superhelical strain in newly synthesized chromatids generated by the DNA translocases SMARCAL1, ZRANB3, and HLTF during limited fork reversal (Tian et al., 2021; Figure 2). Our study also showed that, with replication stress, TOP2A is SUMOylated by the SUMO E3 ligase ZATT, mainly at lysines 1228 and 1240. SUMOylated TOP2A then recruits the SUMO-targeted DNA translocase PICH to stalled replication forks, where PICH branch migrates the Holliday junction structures and drives extensive replication fork reversal (Tian et al., 2021; Figure 2). Based on these findings, we proposed that replication fork reversal has two distinct stages, namely initiation and extension stages (Tian et al., 2021; Figure 2). Like SMARCAL1, ZRANB3, and HLTF, PICH is also a member of the SNF2 family (Figure 1). However, in contrast to SMARCAL1, ZRANB3, and HLTF, PICH possesses branch migration activity but not fork regression activity, indicating that PICH is specifically involved in the extension stage of replication fork reversal.

## Mechanisms for Replication Fork Maintenance and Stability

Under replication stress, the replication fork reverses to form a four-way Holliday junction structure, as discussed above. However, the nascent strands in this structure resemble a one-ended DNA DSB, which is susceptible to nucleases such as MRE11, EXO1 (exonuclease1), DNA2 (DNA replication helicase/nuclease 2), and MUS81 (Thangavel et al., 2015; Lemacon et al., 2017; Mijic et al., 2017). To prevent excessive degradation at stalled forks, the nucleolytic activity of these enzymes has to be regulated accurately. Recent studies have identified several protective mechanisms that maintain replication fork structure and confer genomic stability.

## BRCA1/2 and RAD51

BRCA1/2-mediated stable RAD51 filament formation is required for its protective effect on the regressed arm (Carreira and Kowalczykowski, 2011; Schlacher et al., 2012). Consistent with this, wild-type RAD51, but not its DNA-binding mutant RAD51^*T*131*P*^, stably associates with reversed forks and protects them from Mre11-mediated degradation (Kolinjivadi et al., 2017; Mijic et al., 2017). In addition, inhibition of RAD51 DNA-binding and strand exchange activities by the small molecule B02 destabilizes reversed forks, without causing the fork reversal defects observed upon RAD51 depletion (Taglialatela et al., 2017). Moreover, WRNIP1, a member of the AAA + ATPase family, interacts with the BRCA2/RAD51 complex and participates in the stabilization of RAD51 filaments from degradation by MRE11 (Leuzzi et al., 2016). These findings suggest that RAD51 has both a BRCA1/2-independent fork remodeling function and a BRCA1/2-dependent fork-protecting role. However, it is still unclear exactly how RAD51 protects regressed forks from nuclease-mediated degradation. Physical blocking of nuclease binding, or cooperation with other inhibitory proteins, are putative mechanisms. Furthermore, the RAD51 paralogs also participate in replication fork protection against MRE11 over-resection (Somyajit et al., 2015). Whether RAD51 paralogs dampen nucleases via the same mechanism as the BRCA1/2-RAD51 interaction requires further study.

### FA Components

Fanconi anemia is a rare inherited disorder that results from mutations in *FA* genes, which play key roles in DNA replication and repair (Alter, 2014). The FA core complex is an ubiquitin ligase that detects DNA damage and monoubiquitinates the downstream proteins FANCD2 and FANCI to regulate DNA repair of inter-strand crosslinks (ICL) and homologous recombination repair (Nepal et al., 2017; Liu et al., 2020). In addition to its canonical role in ICL repair, several FA proteins stabilize stalled forks. For example, the FA component FANCD2 prevents MRE11-mediated fork over-processing by stabilizing RAD51 nucleofilaments, similarly to BRCA2 (Schlacher et al., 2012; Kim et al., 2015). Interestingly, a recent study demonstrated that the novel protein BOD1L could also protect stalled forks from genome fragility (Higgs et al., 2015). Being downstream of FANCD2/BRCA2, BOD1L maintained fork stability by inhibiting BLM/FBH1 helicases and stabilizing RAD51 nucleoprotein filaments (Higgs et al., 2015). However, unlike FANCD2, BOD1L suppressed DNA2-mediated degradation rather than MRE11-dependent instability (Higgs et al., 2015). It may seem unintuitive that both FANCD2 and BOD1L stabilize RAD51 at sites of replication damage, but they prevent different types of nucleolytic attack. Future research is required to reveal the precise mechanism underlying RAD51 stabilization.

### RecQ Family of DNA Helicases

The RecQ family of DNA helicases, including RECQL1/4/5, WRN (Werner syndrome protein), and BLM (Bloom’s syndrome helicase), have been shown to be important for maintaining genome integrity (Croteau et al., 2014). These proteins are conserved from bacteria to humans, and mutations therein lead to diseases such as Werner syndrome and Bloom syndrome, as well as premature aging, and cancer proneness (Mojumdar, 2020). Since Werner and Bloom syndromes are both characterized by chromosome fragility and increased cancer predisposition, many studies have investigated whether the Bloom syndrome helicase BLM and Werner syndrome helicase WRN play roles in protecting stalled replication forks.

A previous study found that WRN helicase and exonuclease catalytic activities were needed to prevent MUS81-mediated breakage after HU-induced replication fork stalling (Murfuni et al., 2012). However, that study did not reveal how the different enzymatic activities of WRN collaborate at stalled forks. A more recent finding suggested that WRN exonuclease prevented MRE11/EXO1-dependent over-resection at nascent strands, while its helicase ensured the necessary exonucleolytic processing (Iannascoli et al., 2015). A non-enzymatic function of WRN was also reported (Su et al., 2014). The authors found that WRN could limit MRE11 exonuclease activity and prevent excessive degradation on nascent strands, possibly by stabilizing RAD51 (Su et al., 2014).

Bloom’s syndrome helicase, another RecQ helicase, has also been implicated in replication fork protection upon replication stress. It was reported that BLM and FANCD2 co-localized at stalled forks in response to replication fork stalling agents (Pichierri et al., 2004). Moreover, the FA pathway was shown to be essential for BLM phosphorylation and assembly in nuclear foci in response to DNA interstrand crosslinking agents (Pichierri et al., 2004). Surprisingly, a recent study found that BLM helicase activity was also indispensable for FANCM recruitment and function at stalled forks (Ling et al., 2016). Therefore, it is reasonable to hypothesize that BLM and the FA pathway form a positive feedback loop to ensure sufficient protection of the stalled forks.

Other proteins, such as ABRO1, PALB2, and WRNIP, have also been implicated in stalled replication fork protection (Murphy et al., 2014; Liao et al., 2018; Bennett et al., 2020; Berti et al., 2020a). However, it is not clear how these factors interact in this process, or how they function in response to different replication obstacles.

## Concluding Remarks

Recent studies have raised many questions about fork remodeling caused by replication stress. Although there are various well-established models of fork reversal and remodeling, some questions remain unanswered. For example, on what basis do cells choose one or several of these mechanisms upon encountering a DNA lesion? How do cells recognize and respond to different DNA lesions? How do factors with similar functions work in non-redundant ways? If helicase and polymerase are dissociated during fork reversal, how is the replisome reloaded onto the replication fork when the fork is restarted? Are other factors vital in the balance between fork reversal and restart? We believe that recent progress in our understanding of fork plasticity under genotoxic stress will spark interest in addressing these questions and clarifying the mechanistic link between fork remodeling and genomic instability. In turn, this should lead to a better understanding of the mechanisms underlying replication and the dynamic relationships among the involved processes, thereby leading to more efficient cancer therapies.

## Author Contributions

SQ and GJ wrote the manuscript. JH and LC reviewed and edited the manuscript. All authors contributed to the article and approved the submitted version.

## Conflict of Interest

The authors declare that the research was conducted in the absence of any commercial or financial relationships that could be construed as a potential conflict of interest.
